# Next-Generation Sequencing (NGS)-Based Preimplantation Genetic Testing for Aneuploidy (PGT-A) of Trophectoderm Biopsy for Recurrent Implantation Failure (RIF) Patients: a Retrospective Study

**DOI:** 10.1007/s43032-021-00519-0

**Published:** 2021-03-11

**Authors:** Jing Tong, Yichao Niu, Anran Wan, Ting Zhang

**Affiliations:** 1 Center for Reproductive Medicine, Ren Ji Hospital, School of Medicine, Shanghai Jiao Tong University, Shanghai, 200135 China; 2 Shanghai Key Laboratory for Assisted Reproduction and Reproductive Genetics, Shanghai, 200135 China

**Keywords:** Next-generation sequencing, Preimplantation genetic testing for aneuploidy, Recurrent implantation failure, Trophectoderm biopsy

## Abstract

Recurrent implantation failure (RIF) is an intrigue condition during in vitro fertilization (IVF) cycles or intracytoplasmic sperm injection (ICSI) treatments. The purpose of this retrospective study is to explore the value of next-generation sequencing (NGS)-based preimplantation genetic testing for aneuploidy (PGT-A) of trophectoderm biopsy in the clinical outcomes for RIF patients with advanced age. A total of 265 RIF patients, who underwent 346 oocyte retrieval cycles and 250 PGT-A cycles, were classified as two groups according to the female age, including < 38 and ≥ 38 years old groups. The two groups were statistically comparable in baseline characteristics. The component of aneuploid embryos was significantly higher in advanced age group than in younger age group (68.9 vs 39.9%, *P* < 0.001). But there were no statistically significant differences in pregnancy rate (43.5 vs 64.7%), clinical pregnancy rate (39.1 vs 48.0%), implantation rate (39.1 vs 51.0%), and miscarriage rate (4.3 vs 7.8%) per embryo transfer (ET) between the two groups. Results suggest that the embryo-related factor plays a crucial role in RIF. Maternal age does not influence the implantation potential of euploid blastocysts. The NGS-based PGT-A involving trophectoderm biopsy is valuable for RIF patients of advanced age by improving their clinical outcomes. In conclusion, the NGS-based PGT-A involving trophectoderm biopsy may represent a valuable supplement to the current RIF management. Nonetheless, these findings should be further validated in a well-designed randomized controlled trial.

## Introduction

Recurrent implantation failure (RIF) is a kind of intrigue condition that derives from the repetitive unsuccessful in vitro fertilization (IVF) cycles or intracytoplasmic sperm injection (ICSI) treatments. The ESHRE PGD Consortium defined RIF as “more than 3 embryo transfers with high quality embryos or the transfer of no less than 10 embryos in multiple transfers; exact numbers to be determined by each center” in 2005 [1]. Nonetheless, there is no uniform definition for RIF currently, even though many articles on this topic have been published. RIF can be defined as the absence of implantation after two consecutive cycles of IVF, ICSI, or frozen embryo replacement, where the cumulative number of transferred embryos was ≥ 4 for cleavage-stage embryos and ≥ 2 for blastocysts and all embryos were of good quality and at appropriate developmental stage or as the failure to achieve a clinical pregnancy after transfer of at least 4 good-quality embryos in a minimum of three fresh or frozen cycles in a woman under the age of 40 years [2–4].

RIF can be a consequence of embryo-related factors, oocyte or sperm quality, parental chromosomal anomalies, uterine factors, immunological factors, and thrombophilic conditions [5]. The embryo itself is thought to be responsible for 30–50% of RIF [6, 7]. The transfer of chromosomally anomalous embryos will result in failed implantation. The development of genomic technologies has revolutionized our capability to detect various kinds of genetic abnormalities in embryos. Transfer of euploid embryos reduces implantation failures, and RIF is an indication for preimplantation genetic testing for aneuploidy (PGT-A). Testing blastocysts for aneuploidy through next-generation sequencing (NGS) has been introduced into clinical practice recently, yet there is no research on the value of NGS-based PGT-A in RIF management. Therefore, this retrospective study was carried out aiming to clarify the value of NGS-based PGT-A of trophectoderm biopsy in the clinical outcomes for RIF patients and to provide an up-to-date view on RIF management.

PGT-A, originally termed as preimplantation genetic screening (PGS), has been proven to be valuable for detecting oocytes with common chromosomal aneuploidies among IVF patients of advanced maternal age through fluorescent in situ hybridization (FISH) as early as in 1995 [8]. Later in 2003, a research [9] demonstrates that preimplantation genetic diagnosis (PGD) using FISH along with blastocyst transfer improves the implantation failure outcome. Meanwhile, PGD using FISH probes is also found to be associated with the improved outcomes for RIF women under 41 years [10]. Nonetheless, the results obtained across laboratories are largely conflicting. A research conducted in 2008 suggests that PGT-A may not increase the implantation rates among RIF women [11], while another study [12] reports that the young RIF patients cannot benefit from PGT-A. In 2009, the ACOG committee holds the opinion that no existing data support that PGT-A is beneficial for RIF patients [13], which is consistent with the ESHRE PGD consortium best practice guidelines in 2010 [14]. Additionally, two randomized trials in 2013 [15] and one retrospective cohort study in 2017 [16] also reveal that PGS using FISH has no effect on the perinatal outcomes among RIF women.

Theoretically, PGT-A maximizes the chances of delivering a healthy baby for RIF patients through selecting a euploid embryo to transfer. FISH is among the earliest strategies utilized for PGT-A. However, the conclusions vary when a later version of PGT-A, namely, array comparative genomic hybridization (array CGH) technology, emerges. One web-based questionnaire survey with questions related to practices of and views on PGS is carried out in 2017. According to the results, about 32% responders routinely carry out PGS for RIF, and 84% believe that more randomized controlled trials (RCTs) are warranted to support the use of PGS [17]. A pilot study [18] determines that the array CGH-based PGS with single euploid blastocyst transfer is a successful strategy for RIF. Notably, the ARSM committee opinion in 2018 mentions for the first time that PGT-A for prior implantation failure must be addressed by further research [19]. Moreover, a recent study concludes that live birth rate can be improved using array CGH-based PGT-A with blastocysts transfer during the IVF cycles for patients with a high rate of aneuploidy [20].

Typically, the NGS techniques are the latest and most popular technique utilized in PGT laboratories currently, and they have been routinely adopted for testing embryos by massively parallel genome sequencing worldwide [21]. Specifically, NGS allows for identifying and screening embryos with euploidy, aneuploidy and chromosomal mosaicism. Euploidy is a state known as diploid cells contain 46 chromosomes normally, while aneuploidy refers to an altered condition involving a deviation in copy number from multiples of 23. The definition of chromosomal mosaicism is the co-presence of cells with two (or more) different chromosomal constitutions including whole chromosomal mosaicism, segmental chromosomal mosaicism and complex abnormal mosaicism [22, 23]. The NGS-based PGS dramatically improves the IVF pregnancy outcomes compared with array CGH-based PGS [24].

Variations in the employed biopsy and genetic techniques will lead to intercenter differences concerning the IVF clinical outcomes. As a field, PGT has definitely moved away from the biopsied embryos at the cleavage stage. Increasingly, embryo trophectoderm is being biopsied at the blastocyst stage of the 5- or 6-day-old embryo. The techniques of NGS-based PGD involving cleavage-stage biopsy and fresh embryo transfer have enhanced the clinical pregnancy rate per transfer and the implantation rate for RIF [25]. These encouraging views argue that NGS may represent a valuable supplement to the current aneuploidy screening approaches for RIF. However, no existing study is available to evaluate the usefulness of NGS-based PGT-A of trophectoderm biopsy for RIF so far.

## Methods

### Study Population

**Fig. 1:**
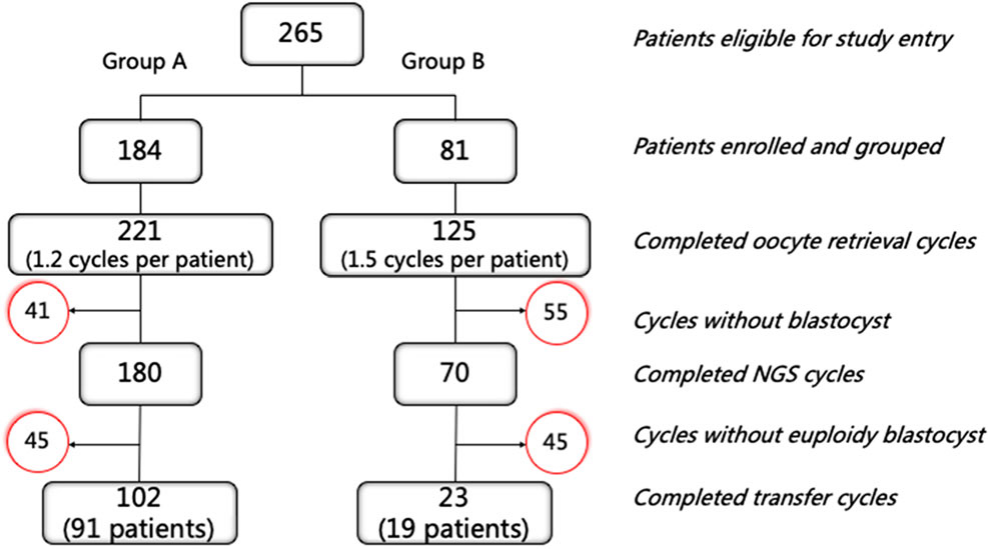
Flow chart of the study process

A total of 265 couples with a history of RIF were recruited into this retrospective study. In our program, RIF was defined as the absence of implantation after two consecutive cycles of IVF, ICSI or frozen embryo replacement, where the cumulative number of transferred embryos was ≥ 4 for cleavage-stage embryos and ≥ 2 for blastocysts, and all embryos were of good quality and at appropriate developmental stage [2]. All couples underwent 346 oocytes retrieval cycles at the Center for Reproductive Medicine of Ren Ji Hospital, Shanghai Jiao Tong University School of Medicine, from August 2018 to September 2019. Thereafter, the study population was divided into two groups according to maternal age. Group A included 184 patients aged < 38 years (mean, 32.8; range, 25-37 years) who completed 221 oocyte retrieval cycles (average, 1.2 cycles per patient) and 180 NGS cycles, and 91 patients had completed 102 transfer cycles at the time of manuscript writing. Group B recruited 81 couples aged ≥ 38 years (mean, 41.3; range, 38–47 years) undergoing 125 oocyte retrieval cycles (average, 1.5 cycles per patient) and 70 NGS cycles, and 19 patients had completed 23 transfer cycles at the time of manuscript writing (Fig. 1). The data collection of this retrospective study was approved by the Shanghai Jiaotong University School of Medicine, Renji Hospital Ethics Committee.

### Ovarian Stimulation and ICSI Protocol

The ovarian stimulation protocol using gonadotrophin-releasing hormone (GnRH) analogues and gonadotrophins depended on patient age and ovarian response. Transvaginal follicular aspiration was carried out at 34–36 h after human chorionic gonadotropin (HCG) injection. Moreover, ICSI was performed to ensure the high fertilization rates and to avoid any contamination caused by the attachment of residual sperm-derived DNA to the zona pellucida at biopsy. Fertilization was assessed at 17–20 h later, which was considered normal when two distinct pronuclei were visible.

### PGT-A Procedure (NGS) and Embryo Transfer

Embryos were cultured to the blastocyst stage under assisted hatching, followed by trophectoderm biopsy on day 5 or day 6. Later, DNA was extracted from the trophectoderm biopsy specimens. UV spectrophotometry was adopted to assess the DNA purity, while the fluorometric methods were utilized to quantify the nucleic acid. The sequencing libraries were typically created by fragmenting DNA and adding specialized adapters to both ends. Afterwards, the prepared libraries were sequenced using Illumina sequencing-by-synthesis chemistry. Then, the data were analyzed, and the sequencing reads were aligned to a reference genome following the Illumina NGS platform protocol. Under a finest background, the NGS platform can distinguish samples with aneuploidy between 20 and 50%. Thus, the embryos with aneuploid percentage between 20 and 50% was classified as mosaic, with aneuploid percentage under 20% was classified as euploid and with aneuploid percentage over 50% as aneuploid [23].

For the sake of cost-effectiveness, no more than 6 embryos were analyzed at one time for each patient. Only patients obtaining euploid embryos were eligible for transfer. Our policy was to transfer one euploid embryo per patient in a hormone replacement cycle by the Guardia^TM^ Access Nano embryo transfer catheter (COOK Medical, Bloomington, Indiana, USA). All patients received progesterone supplementation after embryo transfer, and luteal support was continued for pregnant cases until 12 weeks of gestation.

### Outcome Assessment and Statistical Analysis

Pregnancy was diagnosed based on the rising serum HCG concentration at 14 days after embryo transfer. Clinical pregnancy rate (PR) per transfer was calculated as the percentage of clinical pregnancies with a fetal heartbeat. Implantation rate (IR) was deemed as the percentage of transferred embryos that developed into an implanted gestational sac. Miscarriage rate (MR) was defined as the percentage of clinical pregnancies that were spontaneously miscarried before 20 weeks of gestation.

Differences in basic characteristics and clinical outcomes between two groups were compared through two-sample *t* test, chi-squared or Fisher’s exact test for their significance. All data were analyzed using the SPSS software (version 26.0, IBM Corporation, USA), and *P* < 0.05 indicated statistically significant difference.

## Results

Altogether 265 RIF patients completed 346 oocyte retrieval cycles in this study; of them, trophectoderm biopsies were available in 250 cycles. One hundred ten (41.5%) of those patients analyzed had received euploid embryos, and 125 transfer cycles were completed at the time of manuscript writing. Meanwhile, the pregnancy rate, clinical pregnancy rate, implantation rate, and miscarriage rate per transfer were 57.6, 46.4, 48.8, and 7.2%, respectively.

The demographic and baseline characteristics between two study groups (< 38 and ≥ 38 years) were comparable, including duration and cause of infertility, primary infertility or secondary infertility, baseline concentrations of follicle stimulating hormone (FSH), luteinizing hormone (LH), estradiol, and thyroid stimulating hormone (TSH) before treatment. However, differences in anti-mullerian hormone (AMH) and body mass index (BMI) resulted from age difference were of statistical significance between two groups (Table 1). As expected, the advanced age group had significantly shorter duration of stimulation, less oocytes retrieved, less MII oocytes, less oocytes fertilized, less fertilized oocytes that cleaved, and less blastocysts than those of the younger age group. No significant difference was detected in the dose of FSH used between two groups (Table 2).

**Table 1:** Baseline patient characteristics

Variable	Group A (*n* = 184)	Group B (*n* = 81)	*P* value
Age (years); mean ± SD (range)	32.8 ± 3.1 (25–37)	41.3 ± 2.3 (38–47)	< 0.001
Duration of infertility (years, mean ± SD)	3.8 ± 2.5	4.7 ± 4.7	NS
Primary infertility (%)	47	40	NS
Cause of infertility (%)			NS
Tubal factor	48	40	
Male factor	14	17	
Anovulation	23	17	
Other *	15	26	
AMH (mean ± SD) (ng/ml)	3.8 ± 2.9	1.8 ± 1.2	< 0.001
Basal FSH concentration (mean ± SD) (IU/L)	6.4 ± 3.4	7.0 ± 5.1	NS
Basal LH concentration (mean ± SD) (IU/L)	4.7 ± 3.1	4.0 ± 3.2	NS
Basal estradiol concentration (mean ± SD) (pg/mL)	38.2 ± 22.3	42.8 ± 34.8	NS
TSH (mean ± SD) (μIU/mL)	1.9 ± 1.4	2.1 ± 1.2	NS
BMI (mean ± SD) (Kg/m^2^)	21.6 ± 2.8	22.7 ± 2.6	0.004

*NS*, not statistically significantly different; ^*^, Endometriosis, unexplained factor, combined factors

**Table 2:** Stimulation and PGT-aneuploidy screening data

Variable	Group A	Group B	*P* value
No. of stimulation cycles	221	125	
Duration of stimulation (days)	8.5 ± 1.2	7.1 ± 3.1	< 0.001
Dose of FSH used	1120.0 ± 790.7	962.8 ± 866.1	NS
No. of oocytes retrieved	10.5 ± 6.6	4.9 ± 4.4	< 0.001
No. of MII oocytes	8.5 ± 5.7	4.2 ± 3.9	< 0.001
No. of oocytes fertilized (2PN)	6.7 ± 4.8	3.2 ± 3.0	< 0.001
No. of fertilized oocytes that cleaved	6.6 ± 4.7	3.1 ± 3.0	< 0.001
No. of blastocysts	3.0 ± 2.9	1.1 ± 1.4	< 0.001
PGT-A results			< 0.001
Euploid % (n)	50.8 (300)	25.9 (35)	
Aneuploid % (n)	39.9 (236)	68.9 (93)	
Mosaicism % (n)	9.3 (55)	5.2 (7)	

*NS*, not statistically significantly different

On the other hand, the component of aneuploid embryos was significantly higher in advanced age group than in younger age group (68.9 vs 39.9%, *P* < 0.001). Additionally, the components of euploid and mosaic embryos were remarkably lower in advanced age group than in younger age group (25.9 vs 50.8%, 5.2 vs 9.3%, respectively, *P* < 0.001) (Table 2). Patients in advanced age group obtained an average of 0.43 (35/81) embryos eligible for transfer only, while those in younger age group received an average of 1.63 (300/184) embryos for transfer. However, differences in pregnancy rate (43.5 vs 64.7%), clinical pregnancy rate (39.1 vs 48.0%), implantation rate (39.1 vs 51.0%), and miscarriage rate (4.3 vs 7.8%) per transfer between two groups were not statistically significant (Table 3).

**Table 3:** Clinical outcome following NGS based PGT-A for RIF

Variable	Group A	Group B	*P* value
No. of patients obtained euploidy embryo	91	19	
No. of transfer cycles	102	23	
Pregnancy rate per transfer % (n)	64.7 (66)	43.5 (10)	NS
Clinical pregnancy rate per transfer % (n)	48.0 (49)	39.1 (9)	NS
Implantation rate per transfer % (n)	51.0 (52)	39.1 (9)	NS
Miscarriage rate per transfer % (n)	7.8 (8)	4.3 (1)	NS

*NS* not statistically significantly different

## Discussion

In the absence of studies on the value of NGS-based PGT-A of trophectoderm biopsy in RIF, we conducted this retrospective study—aimed at assessing the role of embryo factors in RIF and thereby clarifying the value of avoiding embryo factors via NGS-based PGT-A in RIF management. In spite of the small incidence of RIF, we still recruited 265 RIF patients in a big reproduction center in China—according to our results—NGS-based PGT-A of trophectoderm biopsy improved the clinical outcomes for RIF patients with advanced age. NGS-based PGT-A of trophectoderm biopsy is the latest, highly accurate, and reliable technology, and it is of crucial importance for RIF management. The embryo-related factors play leading roles in RIF. Notably, these encouraging facts, different from those obtained in former studies, mainly attribute to the development of genetic testing and embryo biopsy technologies.

Chromosomal abnormalities arise during the development of germ cells and/or preimplantation embryos as the maternal age increases [26]. The aneuploid rate seems to reach 30–40% in blastocysts even in women aged less than 35 years [27]. Compared with patients with normal fertility, those with previous IVF failures are associated with a significantly higher age-independent aneuploidy rate [28]. Moreover, plenty of studies reveal that the euploid rate of RIF patients is lower than that in other infertile patients. The embryonic ploidy directly affects the embryo implantation and the successful development of those embryos into healthy babies. Hence, aneuploidy is a leading cause of RIF [9, 18, 29]. Based on our data, the euploid rates were 50.8 and 25.9% in younger and advanced RIF patients, respectively. And there is no difference in rates of pregnancy, clinical pregnancy, implantation and miscarriage between advanced age RIF patients and younger age RIF patients. Maternal age does not influence the implantation potential of euploid blastocysts [30, 31].

Traditionally, embryos for transfer are selected based on the morphology alone. However, morphology is a poor predictor of embryo euploidy, while aneuploidy often shows no morphological manifestation, and the chromosomally chaotic embryos may appear normal morphologically [32]. Moreover, the observation of characteristics is subjective, which exhibits high intrinsic interoperator and intraoperator variations. The PGT-A technology has been developed to improve the assisted-reproduction outcomes by distinguishing euploid embryos from those with lethal aneuploidy. The high aneuploidy frequency in RIF patients, together with the poor predicting capacity of traditional morphology, has promoted the introduction of PGT-A in RIF management by determining embryo euploidy before transfer to the uterus.

Biopsy at advanced stage of embryonic development (like trophectoderm biopsy) is more resilient to technical manipulation than biopsy at cleavage stage. In addition, blastocysts are robust compared with those at earlier embryonic stages, which can better tolerate the insult of biopsy than the cleavage-stage embryos. Trophectoderm biopsy is advantageous, since no cell from the inner cell mass is extracted. Almost 100% blastocysts survive the embryo biopsy, consequently achieving a high embryo implantation rate [33]. Furthermore, multiple cells (such as approximately 3-10 cells) are removed during biopsy at the blastocyst stage, which results in an overall improved accuracy of PGT [21].

Specifically, the chromosomally normal embryos are linked with the highest implantation potential, whereas the chromosomally abnormal ones have the lowest potential. FISH is the first molecular cytogenetic technique used to analyze the interphase nuclei spreading on slides, but it only analyzes a limited number of chromosomes. Moreover, the associated technical difficulties have caused a broad range of error rates between laboratories [34]. Therefore, most researchers conclude that RIF patients cannot benefit from the FISH-based PGT-A. By assessing the entire chromosomal complement of embryo, array CGH detects approximately 42% more abnormalities and 13% more abnormal embryos than those of the standard 12-probe FISH approach [35]. Notably, such more reliable technology is highly specific, with only 1.9% error rate [35], and it brings controversial conclusions regarding RIF management. Nonetheless, a few studies discover that array CGH-based PGT-A is a successful strategy for RIF.

The advances in genetic testing technology undoubtedly increase the scope and efficiency of genetic testing for human embryos, which may bring bright future for RIF management. NGS, the up-to-date technology, has a higher dynamic range than array CGH; as a result, it provides a greater level of resolution and is considered by some studies as the gold standard PGT-A technology due to its high accuracy and reliability [29, 36]. Using the NGS approaches, segmental mosaicism can be detected, and small chromosome deletions or duplications (typically >10 Mb) are also identifiable using such techniques. The mosaicism rate varies widely in literature reports, which ranges from as low as 2% to as high as 40% at blastocyst stage using NGS methods. The vast majority of clinics report that mosaic embryos represent 5–10% of those tested, which is consistent with our data of 9.3 and 5.2% in younger and advanced age groups, respectively. Transfer of mosaic embryos reduces the implantation rate and increases the miscarriage rate [37]. The capability of NGS in detecting mosaicism is stronger than array CGH (20 vs 25%) in a trophectoderm biopsy [23, 38], which greatly prevents the transfer of mosaic embryos that may possibly have a negative effect on IVF outcome. In this study, the encouraging data obtained might be attributed to the advances provided by NGS and trophectoderm biopsy.

Apart from the embryo factors, uterine factors, including polyp, myoma, and adhesion, can also affect the implantation rates. However, such RIF patients were not excluded in this study, which might have minor impact on our results. Similar to other retrospective studies, the patient selection bias was another limitation of this study. In addition, the pregnant patients were not followed up till their labor because of the limited research time. Nevertheless, this study might shed light on further research of RIF management.

In conclusion, this study argues that for RIF patients of advanced age with euploid embryos, the NGS-based PGT-A of trophectoderm biopsy increases the chance of achieving a successful pregnancy. NGS-based PGT-A of trophectoderm biopsy appears to be a reliable management for them. However, these findings should be further validated in a well-designed randomized controlled trial.
